# Social determinants of HIV/AIDS and intimate partner violence: interrogating the role of race, ethnicity and skin color

**DOI:** 10.1590/1518-8345.0000.3280

**Published:** 2020-06-08

**Authors:** Kamila A. Alexander

**Affiliations:** 1 Johns Hopkins University, School of Nursing, Baltimore, MD, United States of America.

**Figure:**
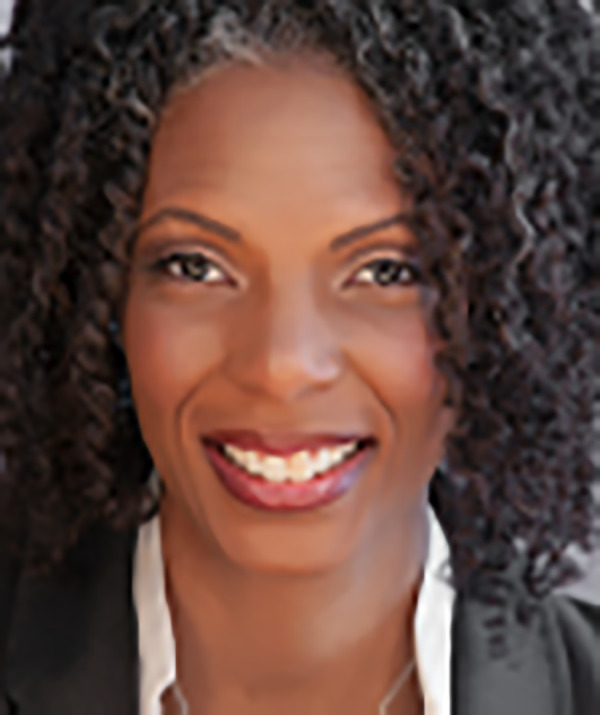


Intimate partner violence (IPV) and HIV/AIDS affect the lives of millions of women across the globe, limiting life expectancy and quality of life. One in three women are affected by IPV. In 2018, worldwide, almost 40 million people were living with HIV. IPV experiences exacerbate risks for HIV/AIDS because people have limited ability to negotiate safe sex, are less likely to use condoms, and are often partnered with people who are engaged in risky behaviors such as drug use and condom-less sexual activity with partners that overlap in time^(^ 1 ^)^. People who are living with HIV (LWH) can experience accelerated disease progression if exposed to IPV, particularly if their partner uses control to interfere with taking life-saving HIV medications or forces sexual activity^(^ 2 ^)^. Unfortunately, IPV and HIV/AIDS disproportionately affect people of the global majority (PGM; also known as people of color) and persons living in poverty.

People with greatest vulnerabilities to IPV and HIV/AIDS are often described by their race, ethnicity, and skin color, perhaps perpetuating false biological or physiological links to disease outcomes. Alternatively, social determinants of health, the conditions in which PGM develop and sustain romantic and sexual relationships, heavily influence transmission of HIV/AIDS and exposure to IPV^(^ 3 ^)^. In this editorial, I offer comments on ways in which two social determinants of health - poverty and diminished gender power are complex constructs through which to interrogate our use of race and gender as intersectional variables in research about these intersecting health epidemics. As an exemplar, I will discuss the impact of HIV/AIDS and IPV on the health of Black women in the U.S.

Despite recent successes in curbing new HIV infections, Black women in the U.S. continue to experience disproportionate effects to health. Almost one-fifth of new HIV infections are Black women and about one in two Black women LWH are affected by IPV. Additionally, about one-quarter of Black women LWH in the U.S. are not engaged in HIV care, and thus also experience poor HIV treatment outcomes^(^ 4 ^)^.

While these facts are discouraging, they cannot be fully understood without careful and nuanced examinations of the underlying causes for these disparities. “Black” (or, in many cases, African-American) and “woman”, as racial and gender categories do not describe a monolithic group of people without individual experiences or specific cultural contexts; yet, as descriptors, they can provide starting points for interrogating the role that social categories of oppression play in our understanding of health inequities and subsequent solutions.

Race, ethnicity, and skin color are often used interchangeably with other markers of marginalization to describe populations most affected by HIV/AIDS and IPV. These characteristics are often aligned with economic disadvantage, cultural, historical, religious, or other social characteristics. Race and skin color, physical superficial characteristics used to group people and ethnicity, shared cultural, linguistic, or country-of-origin hold cultural meanings. In the U.S, people self-identify their race and/or ethnicity; and self-identification is often framed within a society that determines an individual’s value based on these characteristics^(^ 4 ^)^.

Black women in the U.S. often manage multiple stressors driven by social determinants of health that are unique to their life circumstances. For example, Black women are more likely to live in poverty than most other women except for Native American women. Historically, government policies limited where Black families could live, where they could obtain an education, and who they could marry. This created tight neighborhood networks with many advantages for social connection but without monetary investment, became disorganized due to fewer resources for economic opportunities. Activities that can accelerate HIV/AIDS transmission and IPV such as drug sales and consumption as well as sex work became sustained ways to provide basic family needs^(^ 3 ^)^.

Diminished gender power within sexual relationships and families is another social determinant affecting HIV/AIDS and IPV experiences among Black women. For example, the sexual revolution in the 1960s and 1970s was an empowering vehicle for change among White women that included increased acceptance of sex outside of marriage and control of reproductive decisions. However, Black women’s family structures suffered from instability due to constrained economic control and limited sex partner availability. Rates of incarceration among Black men soared, effectively removing them and their earnings from families. Fewer Black men to accommodate the social and sexual desires of Black women resulted in lower condom use negotiating power and increased numbers of sexual partners. Sexual networks were smaller; augmenting opportunities for sexual transmission of HIV/AIDS within an economically strained environment^(^ 3 ^)^.

While poverty and diminished gender power provide context for understanding the lives of Black women as they relate to HIV/AIDS and IPV, their examination does not present a complete picture - one in which resilience drives Black women’s responses to these social inequities. For example, Black women use IPV safety strategies and build on existing sources of strength to overcome the effects of IPV and HIV.. Women experiencing IPV access sources of strength to gain motivation to change their situation, leave their abuser, or take care of their health. Spirituality/religion or belief in God, self-reliance or belief in oneself, and accessing friends or family, police, IPV organizations, and healthcare^(^ 5 ^)^ elicited feelings of empowerment and resiliency. Additionally, shared identities, unique strategies for coping, and awareness of social and political meaning can preserve Black women’s well-being in the face of adversity. Thus, approaches to HIV prevention and HIV care engagement among Black women experiencing IPV should take into account these important culturally-derived aspects for transforming health.

Race, ethnicity, and skin color do not operate alone in our understanding of disparities in health among Black women experiencing IPV. Access to prevention, treatment, care, and support services are essential mechanisms for decreasing the effects of these intersecting epidemics on large populations of people. Previous research and programs approached prevention and treatment by focusing on individual behaviors. However, current practice and research approaches require a broadened lens to include multi-level, culturally and socially-relevant responses that decrease the burden of HIV/AIDS and IPV on populations that are most vulnerable to their effects.
